# Colon-targeted pH-responsive tofacitinib beads improve ulcerative colitis outcomes with lower systemic exposure

**DOI:** 10.1007/s44446-026-00076-0

**Published:** 2026-04-01

**Authors:** Ibrahim M. Ibrahim, Ola Qadi, Osama A. A. Ahmed, Fatemah Kamel, Rania Magadmi, Sameer Alharthi

**Affiliations:** 1 Department of Clinical Pharmacology, Faculty of Medicine, King Abdulaziz University, Jeddah, Saudi Arabia; 2 Pharmacy, Al-Hada Armed Forces Hospital, Taif, Saudi Arabia; 3 Pharmacy Program, Department of Pharmaceutical Sciences, Batterjee Medical College, 21442 Jeddah, Saudi Arabia; 4 Inflammatory Bowel Disease Research Group, King Abdulaziz University, Jeddah, Saudi Arabia

**Keywords:** 2 alginates, 3 colitis, 4 cytokines, 5 tofacitinib, 6 targeted delivery

## Abstract

Ulcerative colitis (UC) is a chronic inflammatory bowel disease resulting in mucosal inflammation and ulceration. Although tofacitinib, a Janus kinase inhibitor, is effective when administered systemically, its clinical use may be limited by systemic adverse effects. Thus, this study aimed to design and evaluate a colon‑targeted tofacitinib delivery system based on sodium alginate beads coated with Eudragit® S‑100 in a dextran sulfate sodium (DSS) induced colitis rat model. Tofacitinib was encapsulated within alginate beads and subsequently coated with Eudragit® S-100 for pH-dependent release. The beads were characterized for size, encapsulation efficiency, stability, and *in vitro* drug release. Therapeutic efficacy was evaluated in a 30-day DSS colitis model by histopathology, cytokine profiling, and drug concentrations in plasma and colon tissue. The colon-targeted beads exhibited minimal release under acidic gastric conditions and maximum release at the colonic pH. *In vivo*, the formulation significantly improved disease activity scores, preserved colonic architecture, and significantly reduced pro-inflammatory cytokines levels while increasing IL-10 levels. Colonic drug levels achieved with the formulated beads were substantially higher than those obtained with non‑formulated tofacitinib, accompanied by markedly reduced systemic exposure. As a conclusion, the colon-targeted tofacitinib delivery system may limit systemic exposure relative to conventional oral administration while maintaining therapeutic efficacy in experimental colitis. Nevertheless, comprehensive toxicological and long‑term safety studies are required before definitive conclusions regarding safety can be drawn.

## Introduction

Ulcerative colitis (UC) is a chronic, relapsing, and remitting inflammatory bowel disease that primarily affects the mucosal layers of the colon and rectum (Villanacci et al. 2025). Histologically, UC is characterized by the identification by mucosal ulceration, crypt distortion, and inflammatory infiltration, while clinically it presents with abdominal pain, bloody diarrhea, urgency, and rectal bleeding (Neurath 2014; Kobayashi et al. 2020). The disease typically begins in the rectum and may extend proximally in a continuous manner, with periods of remission during which symptoms partially or completely subside. The etiology of UC remains undetermined but is believed to involve complex interactions among genetic, environmental, and immunological factors.

Tofacitinib is an oral Janus kinase (JAK) inhibitor that modulates the JAK/STAT signaling pathway, thereby regulating multiple inflammatory cytokines implicated in UC pathogenesis. Clinical studies have demonstrated its efficacy in inducing and maintaining remission in patients with moderate to severe UC, particularly in those who had failed prior biologic therapy (Sandborn et al. 2017). Long‑term follow‑up studies further indicate that a substantial proportion of patients can achieve steroid-free remission by 78 weeks (Carbonnel et al. 2016). However, systemic exposure to tofacitinib is associated with dose-dependent adverse effects, including infections, lipid abnormalities, and thromboembolic events (Winthrop et al. 2018). These safety concerns highlight the importance of site-specific drug delivery with the intent to minimize exposure while preserving therapeutic effects.

Colon-targeted drug delivery systems offer a promising approach to address this limitation. Such systems utilize physiological characteristics of the gastrointestinal tract, including pH variation and microbial activity, to achieve site‑specific drug release (Lee et al. 2020). Among these strategies, pH-dependent delivery using Eudragit® S-100, a methacrylic acid copolymer that dissolves above pH 7, is widely used for colonic targeting (Philip and Philip 2010). This approach enables preferential drug release in the distal intestine, where UC inflammation is typically most pronounced.

Sodium alginate, a natural biopolymer derived from brown seaweed, is frequently used in oral drug delivery due to its biocompatibility, mucoadhesiveness, and ability to form hydrogels upon crosslinking with divalent cations such as calcium (Kothale et al. 2020). Alginate beads can encapsulate therapeutic agents and provide controlled release profiles. When further coated with Eudragit® S-100, alginate‑based systems can restrict premature drug release in the upper gastrointestinal tract and promote targeted delivery to the colon (Suksamran et al. 2009; Chawla et al. 2012).

The efficacy of such colon-targeted formulations has been demonstrated in several preclinical studies. For instance, Eudragit‑coated alginate beads containing curcumin or mesalamine exhibited enhanced colonic drug delivery and improved anti‑inflammatory effects in experimental colitis models (Mutalik et al. 2016). More recently, ileocolonic‑targeted tofacitinib capsules were shown to achieve higher colonic drug concentrations with reduced systemic exposure, supporting the feasibility of site‑specific delivery strategies for JAK inhibitors (Yadav et al. 2022).

Experimental evaluation of colon‑targeted delivery systems is commonly performed using dextran sodium sulfate (DSS)-induced colitis in rodents, a well-established model that reproduces key immunopathological and histological features of human UC. DSS disrupts the intestinal epithelial barrier, promotes inflammatory cytokine release, and results in crypt distortion and mucosal ulceration (Eichele and Kharbanda 2017). Targeted delivery of tofacitinib has been shown to suppress pro-inflammatory cytokines such as interleukin‑6 (IL‑6), and interleukin‑23 (IL‑23), while enhancing anti-inflammatory interleukin‑10 (IL‑10), thereby contributing to mucosal healing (Leach et al. 1999; Ghoreschi et al*.* 2011).

Based on this rationale, the present study aimed to design and evaluate a colon-targeted tofacitinib delivery system using sodium alginate beads coated with Eudragit® S-100. The formulation was first characterized through in vitro drug release studies to confirm pH-dependent release behavior consistent with colonic targeting. Subsequently, its therapeutic efficacy was evaluated in a DSS-induced colitis rat model through histological assessments, cytokine profiling, and quantification of tofacitinib levels in plasma and colon tissues. To enable meaningful interpretation of therapeutic performance and drug distribution, the study incorporated direct in vivo comparison with non‑formulated tofacitinib administered at an equivalent dose, as well as with standard therapy (sulfasalazine). Collectively, this approach was designed to evaluate whether localized colonic delivery could enhance anti‑inflammatory efficacy while reducing systemic drug exposure, thereby addressing a key limitation of current UC therapy.

## Materials and methods

### Reagents and instruments

Tofacitinib was sourced from Xi'an Salus Nutra Bio-Tech Inc. (China), while commercial tofacitinib tablet (Lot number: FM5302) and sulphasalazine were purchased from a local Pharmacy in Saudi Arabia. Dextran sodium sulphate (DSS, MW ~ 40,000) was obtained from Thermo Fisher Scientific (Canada), and Eudragit S-100 from Evonik Industries AG (Germany). Both sodium alginate and calcium chloride were supplied by Sigma-Aldrich (St. Louis, USA). Hematoxylin and eosin (H&E) stains were also obtained from Sigma-Aldrich (USA). Rat IL-6, IL-10, IL-23, and INF-γ enzyme-linked immunosorbent assay (ELISA) kits were purchased from Elabscience Biotechnology Co. (MO, USA). The protein assay kit was sourced from Thermo Fisher Scientific (Australia). Unless otherwise specified, all other chemicals were of high-performance liquid chromatography (HPLC) grade and were used as received.

Quantification of tofacitinib concentrations in plasma and colon tissue was performed using an Agilent 1200 series HPLC system coupled with an Agilent 6460 Triple Quadrupole Mass Spectrometer (Santa Clara, CA, USA). Colon tissue homogenates were prepared using an IKA disperser (Pharmacy College, ––-). Morphological characterization of colon-targeted tofacitinib beads was performed with a Quanta 250 FEI scanning electron microscope (SEM) (––-). In vitro drug release studies were carried out using a USP Dissolution Apparatus II (37 °C, 50 rpm). Paraffin-embedded colon tissues were sectioned at 4 µm thickness using a microtome, stained with hematoxylin and eosin, and morphometric analyses were conducted using Las X Office-Leica Microsystems CMS system (Leica Microsystems, GmbH, Germany).

### Preparation of colon-targeted tofacitinib beads

#### Bead formulation

Colon-targeted tofacitinib beads were prepared according to the method described by Mandal et al. (2010). Briefly, 500 mg of tofacitinib was dispersed in 50 mL of distilled water using a magnetic stirrer at room temperature until complete dispersion, then diluted to a final volume of 100 ml. Sodium alginate (2 g) was added to obtain a 2% w/v sodium alginate-tofacitinib (SALG-tofacitinib) solution. A coagulation fluid was prepared by dissolving 44.1 g of calcium chloride in 800 mL of distilled water to obtain 0.5 M calcium chloride solution. The SALG-tofacitinib solution was extruded through a 20 mL syringe fitted with a 21-gauage needle into the coagulation solution under mechanical stirring at 200 rpm for 30 min. The resulting beads were washed with distilled water, filtered, oven-dried at 40 °C for 24 h, and then air-dried at room temperature for an additional 24 h. Placebo beads were prepared using the same procedure, without the addition of tofacitinib.

#### Eudragit® S-100 coating

A laboratory‑developed modified spray‑coating technique was employed. Dried beads were coated with 5% w/v Eudragit® S‑100 dissolved in acetone. Beads were placed on a stainless‑steel mesh support undergoing oscillation using a vortex mixer (Thermo Scientific™ Digital Vortex Mixer, USA) operating in continuous mode, thereby generating a pseudo‑fluidized state. The coating solution was sprayed evenly while the beads were rotated to ensure uniform coverage, followed by drying at room temperature. The coating process was repeated until an approximate 10% increase in bead weight was achieved.

### Bead characterization

#### Encapsulation efficiency and loading capacity

Crushed beads (67 mg) were dissolved in a formic acid:acetonitrile mixture (20:80 v/v) and agitated for 24 h. Tofacitinib concentration was determined HPLC (Agilent 1200 Series) at 285 nm. Encapsulation Efficiency (EE%) and Loading Capacity (LC%) were calculated using the following equations (El-Say 2016 and Malatani et al. 2023):

$$\text{Drug encapsulation efficiency }\left(\mathrm{EE}\right)\mathrm{\%}= \frac{AQ}{TQ} \times 100$$

$$\text{Loading Capacity }\left(\mathrm{LC}\right)\mathrm{\%}= \frac{Weight of the drug}{Weight of beads} \times 100$$

#### Shape, size and morphology

Bead shape was assessed by visual inspection, and average bead diameter was measured using a caliper. Surface morphology, including the presence of pores, cracks, and surface irregularities, was examined using a scanning electron microscope (SEM; Quanta 250 FEI). Samples were mounted on aluminum stubs using carbon conductive tabs and analyzed under thermionic emission conditions.

#### Stability assessment

Stability studies were conducted by storing the beads in a climatic test chamber (Cavallo 1400CFLU, Italy) maintained at 25 ± 2 °C and 60 ± 5% relative humidity (RH) for five months. Following storage, beads were re‑evaluated for encapsulation efficiency as described in Sect. 2.3.1.

#### *In vitro* drug release study

*In vitro* drug release study was conducted using a USP dissolution apparatus II. For comparison, commercial tofacitinib tablets were finely crushed prior to testing to ensure homogeneous dispersion in the dissolution medium. To evaluate the site-specific release, two different buffer solutions were prepared and used as dissolution media. An intermediate intestinal pH phase (pH 6.8) was excluded, as Eudragit® S‑100 remains insoluble below pH 7, and negligible drug release is expected under such conditions. Beads were initially placed in 0.1 N hydrochloric acid (pH 1.2) for 2 h, followed by transfer to phosphate buffered saline (PBS; pH of 7.1) to mimic the conditions in the stomach and intestine. The release study was carried out for a total of 24 h. The dissolution was carried out using 500 mL of each solution at 50 rpm and 37 °C. Samples (1 mL) were withdrawn at 0, 0.5, 1, 2, 3, 4, and 6-time intervals up to 24 h. The samples were analyzed by HPLC analysis at 285 nm (Kyatham et al. 2024), A fresh buffer solution with the same volume as the withdrawn samples was added each time after withdrawal to maintain a constant volume throughout the release study.

### *In vivo* study design

All *in vivo* experimental procedures were reviewed and approved by the Research Ethics Committee of the Faculty of Pharmacy, King Abdulaziz University (PH‑1444‑21, dated 6 December 2022). The approved protocol, animal housing conditions, and welfare measures remained unchanged throughout the study. All experiments were conducted in accordance with the institutional guidelines for the care and use of laboratory animals and adhered to the National Committee of Bioethics (NCBE) regulations.

**Fig. 1:**
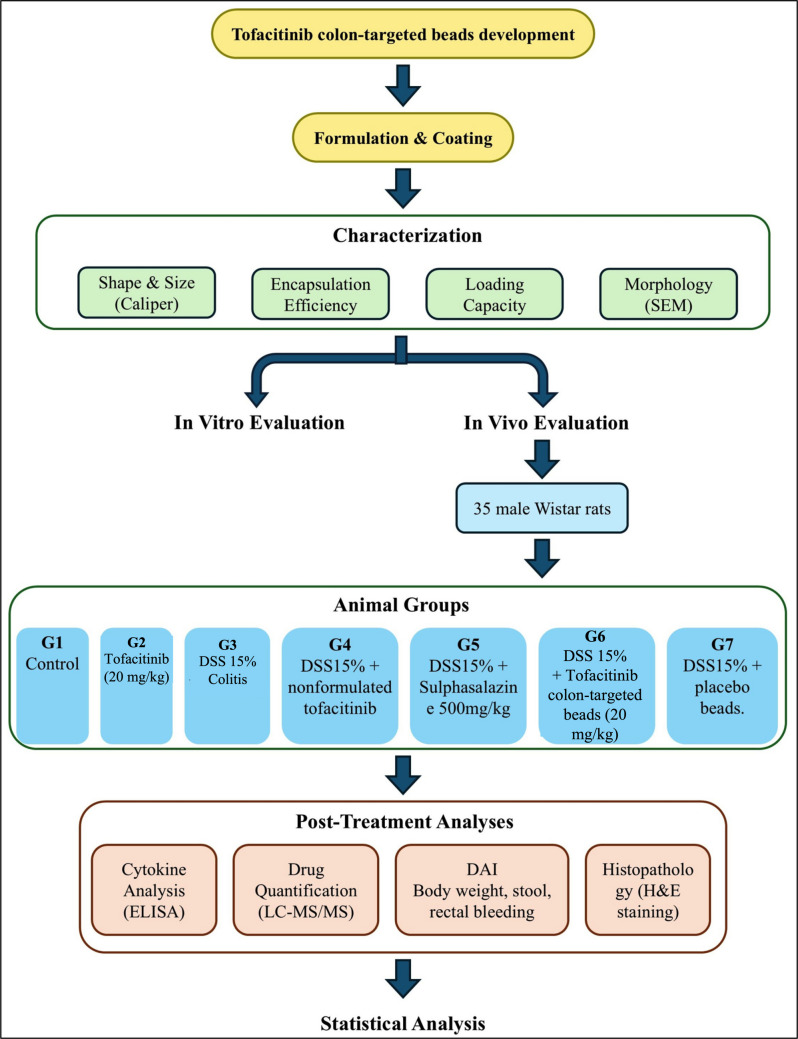
Schematic of the Experimental Design for Evaluating Tofacitinib Formulations in a DSS-Induced Colitis Rat Model. SEM; scanning electron microscopy, DSS; dextran sulfate sodium, ELISA; enzyme‑linked immunosorbent assay, MS; mass spectrometer, DAI; disease activity index, H&E; Hematoxylin and eosin

Thirty-five male Wistar rats (200 ± 20 g) were randomly assigned to seven groups (n = 5/group) and acclimatized for one week in plastic cages (5 rats per cage) under controlled conditions (22–24 °C, 12-h light/dark cycle) with free access to standard rat chow and water. The experimental design is illustrated in Fig. 1. Group 1 (Control): Received 2.5 mL of 0.5% sodium carboxymethylcellulose (NaCMC) orally twice daily. Group 2 (Tof): Received tofacitinib (20 mg/kg) orally twice daily in 0.5% NaCMC, freshly prepared, for 30 days. Group 3 (Colitis): Administered 2.5 mL of 15% dextran sodium sulphate (DSS, MW ∼40,000, CAS 9011–18-1, Cayman Chemicals, USA) orally twice daily for 30 days to induce colitis, based on a pilot study. Group 4 (Colitis + NF-Tof): Received 2.5 mL of 15% DSS solution orally twice daily, followed by tofacitinib (20 mg/kg) twice daily (2 h post-DSS) for 30 days. Group 5 (Colitis + Sulpha): Received 2.5 mL of 15% DSS solution orally twice daily, followed by sulphasalazine (500 mg/kg) twice daily (2 h post-DSS) in 0.5% NaCMC for 30 days. Group 6 (Colitis + Tof-Beads): Received 2.5 mL of 15% DSS solution orally twice daily, followed by tofacitinib colon-targeted beads (20 mg/kg) twice daily (2 h post-DSS) for 30 days. Group 7 (Colitis + Beads): Received 2.5 mL of 15% DSS solution orally twice daily, followed by placebo beads (without tofacitinib) twice daily (2 h post-DSS) for 30 days.

The rats were monitored daily for body weight, stool consistency, and rectal bleeding throughout the study. The disease activity index (DAI) was calculated on days 0, 14, and 30 for statistical analysis (Table 1) (Park et al. 2015). DAI scoring was performed by an investigator blinded to group allocation. A humane endpoint was defined for severe symptoms, including body weight loss greater than 15%, watery diarrhea, or fresh rectal bleeding. Animals were monitored twice daily for signs of discomfort, including reduced mobility, piloerection, or hunched posture. When any rat displayed indications of pain or distress, analgesia was provided using buprenorphine (0.05 mg/kg, subcutaneously) as needed while ensuring it did not interfere with colitis scoring. After the 30-day experimental period, the animals were euthanized via cervical dislocation in accordance with ethical guidelines. Colon tissues were fixed in 10% neutral buffered formalin for histopathological examination, while the remaining tissues were rapidly frozen in liquid nitrogen and stored at − 80 °C for subsequent biochemical analyses.

**Table 1:** Disease activity index (DAI) criteria

Score	Body weight loss (%)	Stool consistency	Fecal blood
0	0	Normal	No blood
1	1–5	Normal, but pale	+
2	5–10	Soft	+ +
3	10–15	Loose/mild diarrhea	+ + +
4	> 15	Watery diarrhea	Fresh rectal bleeding

### Histopathology and morphometry

Distal colon samples were fixed in 10% buffered formalin, processed, and stained with hematoxylin and eosin. Histological evaluation included assessment of crypt architecture and inflammatory infiltration. Morphometric measurements (crypt depth, submucosa, and tunica muscularis thickness) were performed using Leica Las X software. All histological assessments and morphometric measurements were performed by two independent observers who were blinded to the treatment groups.

### Cytokine analysis

Pro‑ and anti‑inflammatory cytokines levels in rat colon tissue homogenates (included IL‑6, IL‑10, IL‑23, and interferon‑γ (IFN‑γ)) were quantified using commercial enzyme‑linked immunosorbent assay (ELISA) kits (Elabscience Biotechnology, MO, USA) according to the manufacturer’s instructions.

Following euthanasia, distal colon segments were excised, rinsed with phosphate‑buffered saline (PBS; pH 7.4), and homogenized in ice‑cold PBS (1:10 w/v). The supernatants obtained after centrifugation were stored at − 80 °C until analysis. Cytokine concentrations were determined from standard curves and normalized to total protein content, measured using the bicinchoninic acid (BCA) assay. Results were expressed as pg per mg of protein.

### Tofacitinib quantification in plasma and colon tissue

Plasma was collected 2 h post-dose via retro-orbital sampling. At the same time point, colon tissues were dissected and homogenized and processed similarly to cytokine assays (Lee & Kim 2019). Samples were extracted using optimized liquid–liquid extraction. Tofacitinib concentrations were analyzed using LC–MS/MS (Agilent 1200 Series HPLC with 6460 Triple Quad MS, Agilent Technologies, Germany). The 2‑hour post‑dose time point was selected to allow comparative assessment of relative colonic and systemic exposure near reported Tmax values rather than full pharmacokinetic profiling.

Chromatographic separation was achieved on an Eclipse Plus C18 column (3.5 µm, 4.6 × 150 mm) at 25 °C. The mobile phase consisted of acetonitrile: methanol: water (containing 0.1% ammonium acetate and 0.1% formic acid) in a 30: 35: 35 (v/v) ratio, delivered isocratically at 0.5 mL/min with a 5 µL injection volume and 15 min run time. Detection was performed in positive electrospray ionization (ESI⁺) mode under multiple reaction monitoring (MRM). Instrument settings were: gas temperature 325 °C, gas flow 10 L/min, nebulizer 35 psi, capillary voltage 4000 V, fragmentor 135 V, and cell accelerator voltage 7 V. Data were processed using MassHunter Workstation software (version B.03.01).

Calibration standards were prepared from methanolic stock solutions of tofacitinib and sildenafil citrate (internal standard, IS) at 1 mg/mL. Serial dilutions of tofacitinib (50–2500 pg/µL) were prepared with a fixed IS concentration of 20 ng/µL. Calibration curves were linear (r^2^ > 0.999) with limits of detection (LOD) and quantification (LOQ) of 15 pg/µL and 50 pg/µL, respectively.

For analysis, plasma was collected 2 h after dosing, centrifuged (3400 rpm, 15 min), and supernatants stored at –80 °C. Colon tissues were washed with cold PBS (pH 7.4), homogenized (1:10 w/v), and centrifuged (5000 rpm, 10 min); the resulting supernatants were similarly stored. Colon tissue concentrations (ng/mL homogenate), corresponding to ng drug per g wet tissue based on the 1:10 (w/v) homogenization ratio. Prior to extraction, recovery procedures were optimized using spiked control samples, achieving a mean recovery of 97 ± 0.7%. For extraction, 100 µL of sample was mixed with 25 µL of 1% ammonia and 50 µL of IS solution, then extracted twice with 3 mL diethyl ether. The combined organic phase was evaporated at 50 °C, reconstituted in methanol, and a 5 µL aliquot was injected into the LC–MS/MS system.

The method was validated according to bioanalytical guidelines. Precision (RSD < 5%) and accuracy (within ± 5%) were achieved over the linear range. Tofacitinib remained stable through three freeze–thaw cycles (< 5% variation). Quantification was based on the ratio of tofacitinib to IS peak areas using the established calibration curve.

### Statistical analysis

Statistical analyses were performed using GraphPad Prism (version 6.01) and IBM SPSS Statistics (version 26). Data are presented as mean ± standard deviation (SD), with the exception of pharmacokinetic (PK) parameters, which exhibited non‑normal distributions and are therefore reported as medians. For the DAI, differences among treatment groups over time were assessed using two‑way repeated‑measures analysis of variance (ANOVA), followed by Tukey’s post‑hoc test. Colon length was evaluated using one‑way ANOVA followed by Tukey’s post‑hoc test. For all other outcomes, including histological scores, cytokine concentrations, and tissue drug levels, group comparisons were performed using the Kruskal–Wallis test, followed by Dunn’s post‑hoc test with appropriate multiple‑comparison adjustment. For tofacitinib concentrations in plasma and colon tissue, which involved non‑parametric comparisons between two independent groups, the Mann–Whitney U test was used. A* P*‑value < 0.05 was considered statistically significant.

## Results

### Colon-targeted beads with high encapsulation and controlled pH-dependent release profile

Tofacitinib-loaded sodium alginate beads were successfully formulated using ionic gelation and subsequently coated with Eudragit® S-100 to achieve colon-targeted drug release. The encapsulation efficiency was 48 ± 1.15%, as determined by HPLC analysis, which showed that 2.4 mg of tofacitinib was encapsulated in a 67 mg bead sample (theoretical content: 5 mg). The loading capacity (LC%) was calculated to be 3.6%. After five months of storage, the EE% remained relatively stable (47.2 ± 0.72%), indicating good formulation stability.

**Fig. 2:**
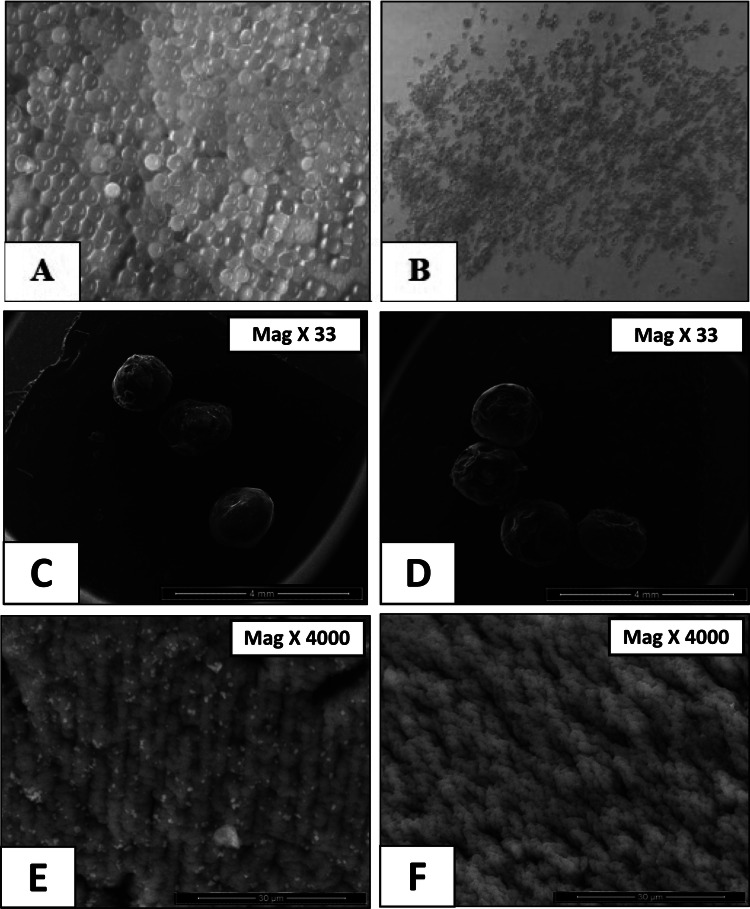
Tofacitinib colon targeted beads/placebo beads. A, before drying; B, after coating and drying; C & E, SEM photo images of tofacitinib colon-targeted beads. D & F, SEM photo images of placebo coated beads

Fresh beads were spherical with an average diameter of 3.5 ± 0.15 mm, which decreased to approximately 1 ± 0.2 mm after drying. (Fig. 2 (A-F)). Morphological examinations by SEM revealed well-defined spherical shapes with intact surfaces. SEM images showed no cracks or pores, and the surface texture remained smooth, suggesting successful Eudragit® S-100 coating.

### Tofacitinib colon-targeted beads showed elevated drug release at pH 7.2

**Fig. 3:**
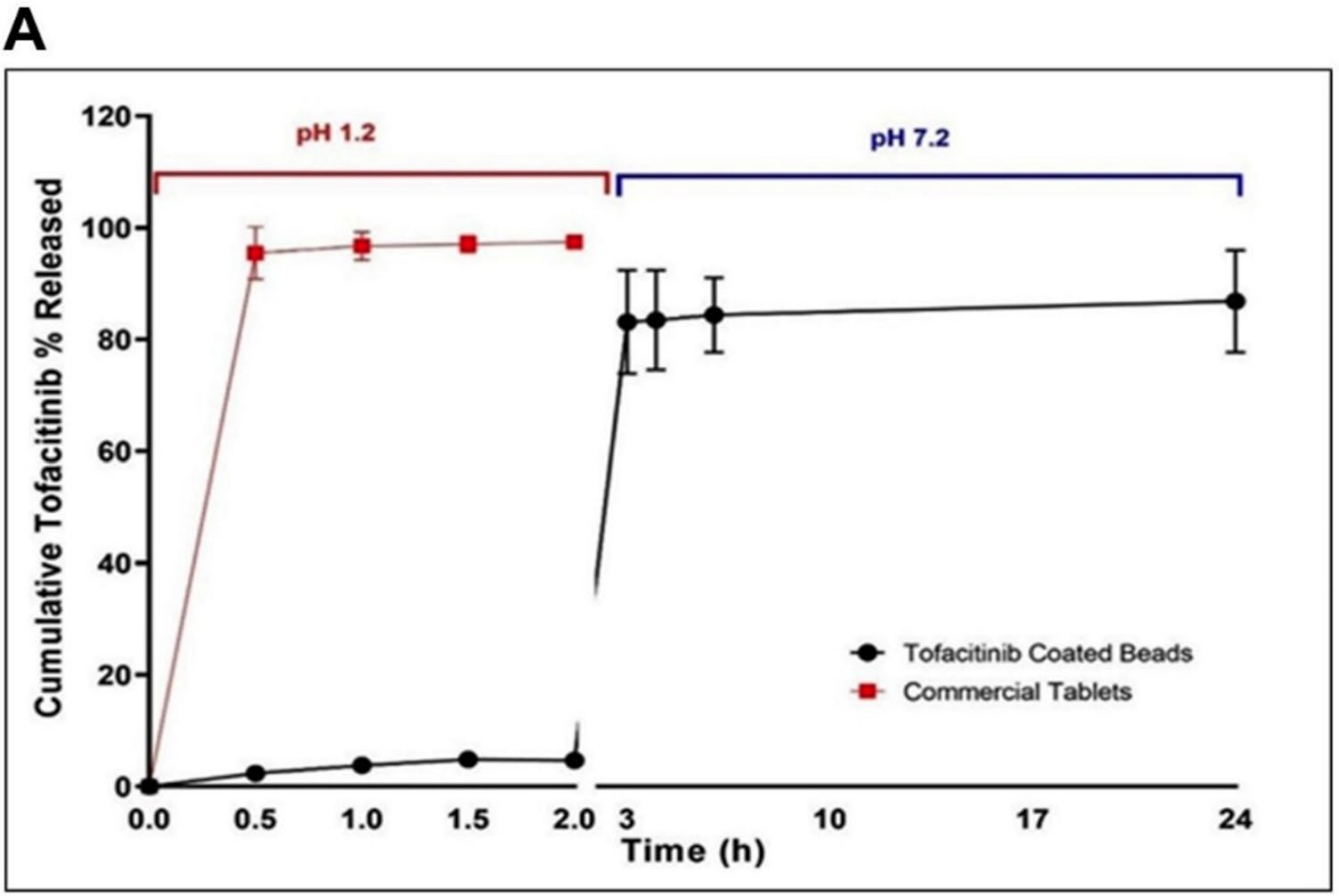
Cumulative release profiles of tofacitinib colon-targeted beads and commercial tofacitinib

*In vitro* drug-release studies were conducted to characterize the release profiles under different pH conditions. In simulated gastric fluid (pH 1.2), only 4.7% of tofacitinib was released after 2 h. Upon transfer to pH 7.2, cumulative drug release markedly increased, reaching 83.09% at 3 h, 83.46% at 4 h, 84.37% at 6 h, and 86.85% at 24 h. These findings demonstrate the pH‑dependent behavior of Eudragit® S‑100 and confirm its suitability for colon‑targeted drug delivery. In contrast, commercially available tofacitinib tablets exhibited rapid burst release in acidic medium, with more than 96% of the drug released within 2 h, indicating non-specific release and a higher systemic exposure risk (Fig. 3).

### Tofacitinib beads prevent DSS-induced colitis

#### Colon-targeted tofacitinib beads prevent DSS-induced colon shortening

**Fig. 4:**
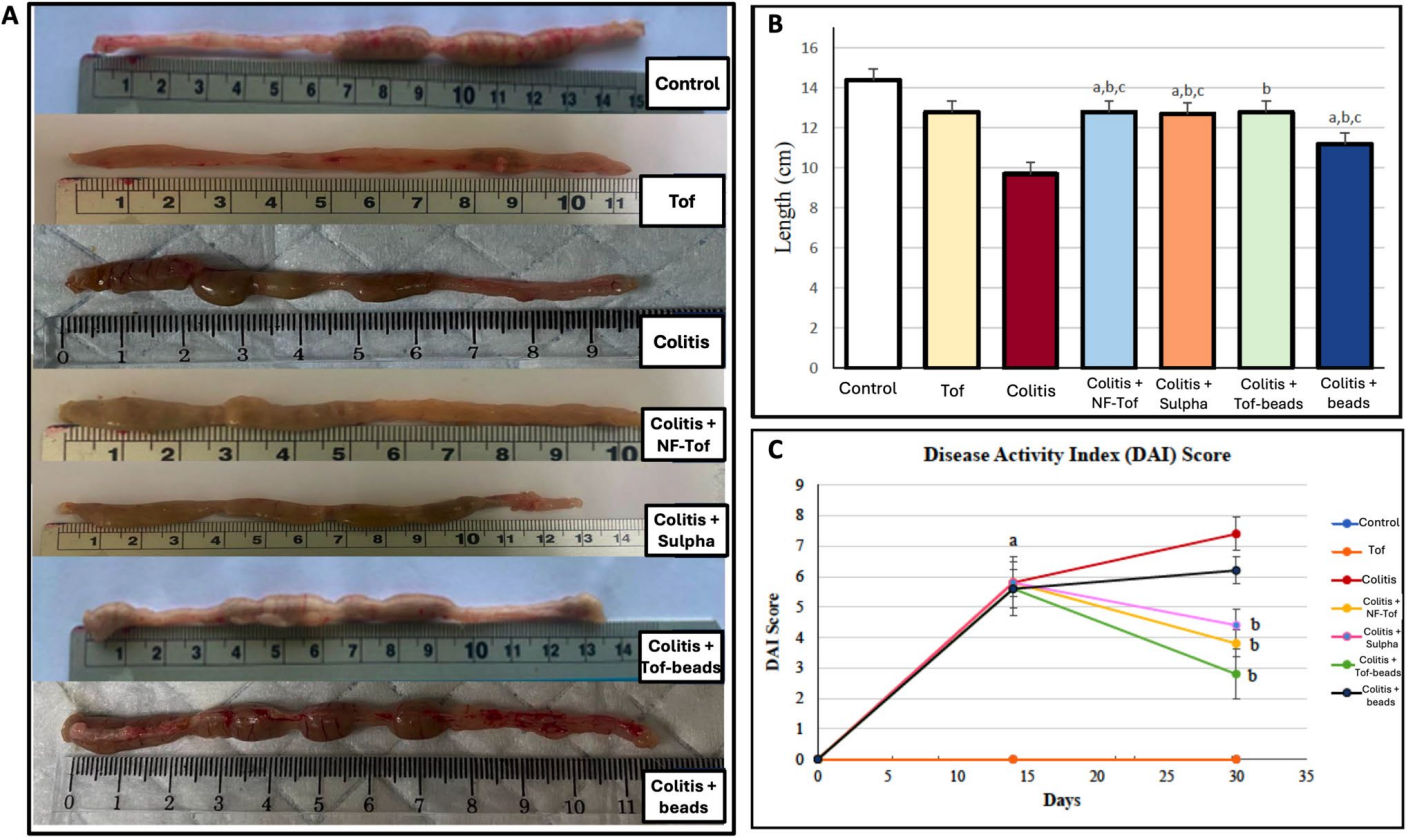
The Effect of different treatments on DSS-Induced Colon Shortening (**A**) Rats colon length in different groups. (**B**) Graphical representation showing comparison of colon length between different groups; (**C**) Disease activity index (DAI) score of different groups, Control group, Tof: Tofacitinib 20 mg/kg group; Colitis group: Colitis + NF-Tof, DSS 15% plus non-formulated tofacitinib 20 mg/kg group; Colitis + Sulpha, DSS 15% plus Sulphasalazine 500 mg/kg; Colitis + Tof-beads: DSS 15% plus tofacitinib colon-targeted beads 20 mg/kg group; and Colitis + beads: DSS 15% plus coated placebo beads group. Statistical analysis for colon length was carried out using one-way ANOVA, followed by Tukey's post-hoc test, while DAI was analyzed using two‑way repeated‑measures ANOVA followed by Tukey’s post‑hoc test. a: Statistically significant from control group values (*P* < 0.05), b: Statistically significant from colitis group values (*P* < 0.05), and c: Statistically significant from tofacitinib colon‑targeted beads group (*P* < 0.05)

Colon shortening is a well-recognized hallmark of DSS-induced colitis. DSS-only rats had significantly shortened colons (9.7 ± 0.44 cm) compared to controls (14.4 ± 0.5 cm). Treatment with colon-targeted tofacitinib beads effectively preserved colon length to 13.8 ± 0.83 cm, compared to control values. This restoration was superior to both non-formulated tofacitinib (12 ± 1 cm) and sulfasalazine (12.6 ± 0.89 cm). Placebo bead treatment resulted in partial preservation (11.2 ± 0.83 cm), which was not statistically significant compared to DSS alone (Fig. 4A and B).

#### Colon-targeted tofacitinib beads significantly reduce disease activity in colitis

The disease activity index (DAI), which integrates weight loss, stool consistency, and rectal bleeding, was markedly elevated in the DSS-only group, reaching 5.80 ± 0.48 on day 14 and 7.40 ± 0.55 on day 30. In contrast, rats treated with colon-targeted tofacitinib beads showed the most significant reduction in DAI by day 30 (2.80 ± 0.48), outperforming both the non-formulated tofacitinib group (3.80 ± 0.44) and the sulfasalazine-treated group (4.40 ± 0.54). The placebo bead group showed a DAI of 6.20 ± 0.45, indicating minimal therapeutic benefit. These results consistently demonstrate the superiority of localized drug delivery in mitigating clinical signs of colitis (Fig. 4C).

### Histological regeneration and inflammation reduction with colon-targeted tofacitinib beads

**Fig. 5:**
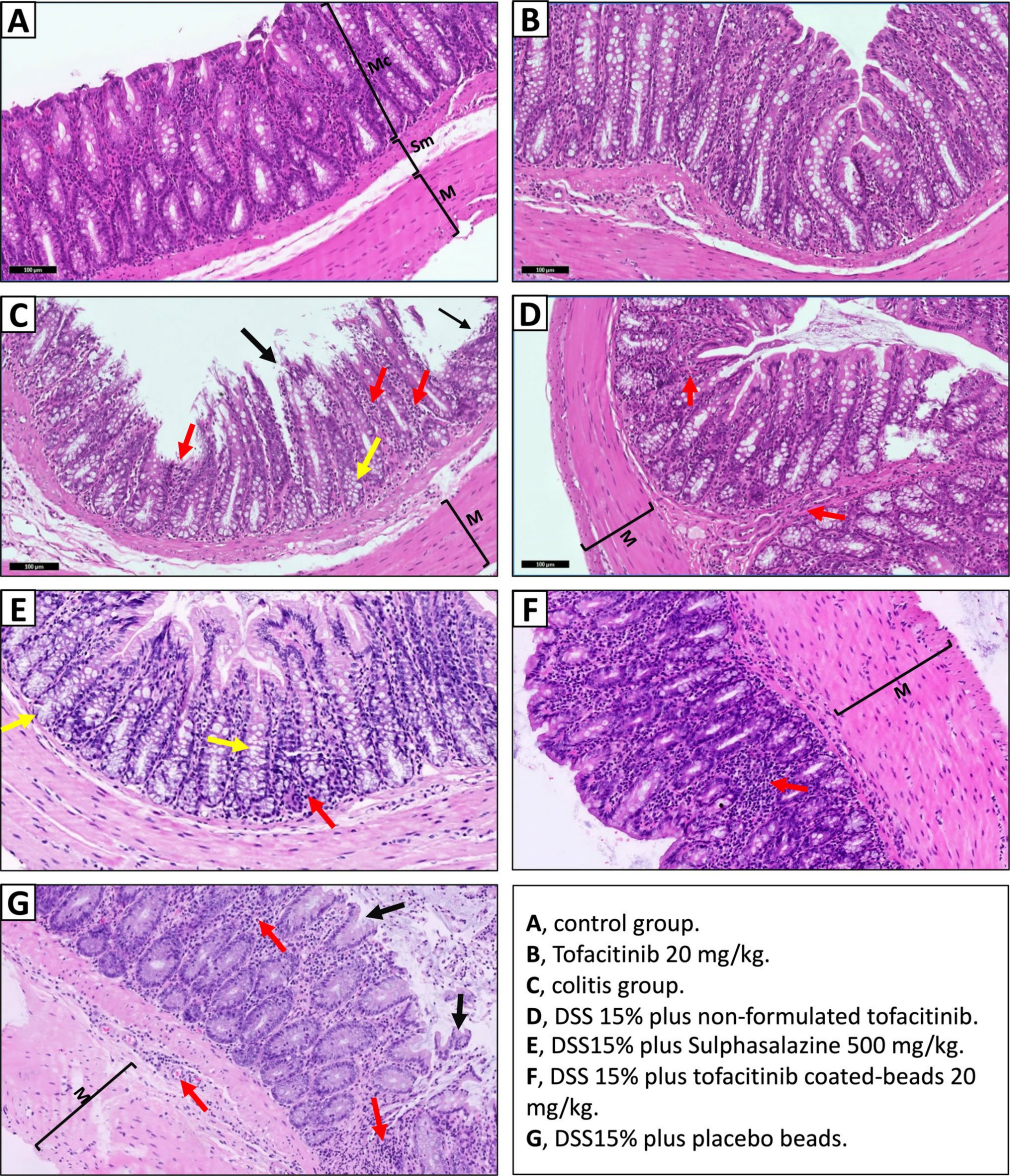
Photomicrographs of rat colon stained with H&E. **A**, control group; **B**, Tofacitinib 20 mg/kg; **C**, colitis group; **D**, DSS 15% plus non-formulated tofacitinib, **E**, DSS15% plus Sulphasalazine 500 mg/kg; **F**, DSS 15% plus tofacitinib coated-beads 20 mg/kg; **G**, DSS15% plus placebo beads. sloughed epithelial cells are marked with black arrows, the inflammatory cells are marked with red arrow, and vacuolar degeneration is marked with yellow arrow. Mc; mucosa, Sm; submucosa, and M; muscularis

The histological examination of colon tissue from the control group showed normal luminal epithelium with no folding (Fig. 5, A). In the Tofacitinib group alone (20 mg/kg), the normal histological architecture of the mucosa, submucosa, and muscularis was preserved, with no signs of inflammation (Fig. 5, B). In contrast, the colitis group exhibited pronounced histopathological alterations (Fig. 5, C), including epithelial sloughing (black arrow), vacuolar degeneration of crypt cells (yellow arrow), inflammatory cells infiltration within the lamina propria (red arrow), and thickening of the tunica muscularis mucosa. In the DSS 15% + non‑formulated tofacitinib 20 mg/kg group, milder inflammatory cell infiltration (red arrow) and cellular degeneration were observed, and the luminal epithelium remained intact (Fig. 5, D). In the DSS 15% + Sulphasalazine 500 mg/kg group (Fig. 5, E), although epithelial sloughing was absent, vacuolar degeneration of cryptal epithelium was pronounced (yellow arrow), with moderate lymphocytic infiltration in submucosa, and marked muscular thickening (red arrow). In DSS 15% + colon-targeted tofacitinib beads group (Fig. 5, F), luminal and glandular epithelia were protected and appeared normal, with minimal leucocytic infiltration (red arrow) and reduced muscular thickening. In the placebo beads group (Fig. 5, G), many luminal epithelial cells were lost (black arrow) while glandular epithelium showed vacuolar degeneration. The submucosa showed marked leucocytic infiltration (red arrow), and marked thickening in tunica muscularis.

### Targeted tofacitinib reverses DSS-induced increases in crypt depth and wall thickness

**Fig. 6:**
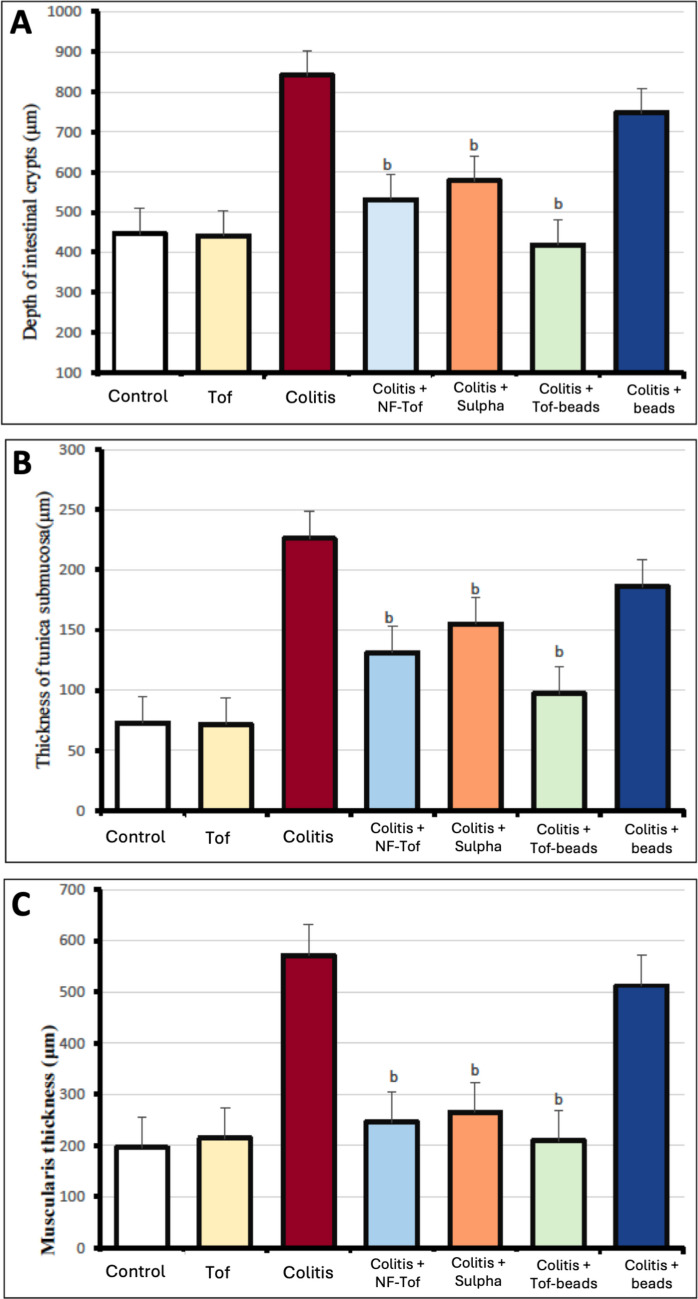
Impact of tofacitinib on DSS-induced increases in crypt depth and wall thickness of colon: (**A**) Depth of intestinal crypts in rat colon tissue; (**B**) Thickness of tunica submucosa of rat colon tissue; (**C**) Thickness of tunica muscularis of rat colon tissue. Control group, Tof: Tofacitinib 20 mg/kg group; Colitis group: Colitis + NF-Tof, DSS 15% plus non-formulated tofacitinib 20 mg/kg group; Colitis + Sulpha, DSS 15% plus Sulphasalazine 500 mg/kg; Colitis + Tof-beads: DSS 15% plus tofacitinib colon-targeted beads 20 mg/kg group; and Colitis + beads: DSS 15% plus coated placebo beads group. Data are expressed as means ± SD. Statistical analysis was carried using the Kruskal–Wallis test, followed by Dunn’s post‑hoc test. a: Statistically significant from control group values, (*P* < 0.05), and b: Statistically significant from colitis group values, (*P* < 0.05)

Quantitative morphometric analysis showed that the intestinal crypts depth in rats treated with tofacitinib alone (20 mg/kg) without DSS 15% was comparable to that of the control group (441.4 ± 36.3 µm vs 446.4 ± 65.1 µm). DSS administration caused a significant 1.9‑fold increase in crypt depth (841.5 ± 67.0 µm, *P* < 0.05 vs. control). Treatment with non‑formulated tofacitinib (531.4 ± 57.9 µm), sulfasalazine group (579.0 ± 55.7 µm) and colon‑targeted tofacitinib beads (418.0 ± 74.2 µm) significantly reduced crypt depth by approximately 37%, 31%, and 50%, respectively, compared to the DSS group (*P* < 0.05). The colon‑targeted formulation produced the greatest improvement, restoring crypt depth close to normal levels. In contrast, the placebo bead group (747.7 ± 64.2 µm) showed no significant improvement relative to the DSS group (Fig. 6 (A)).

In control rats, the tunica submucosa measured 73.0 ± 14.6 µm, with comparable values observed in the tofacitinib‑alone group (71.6 ± 9.3 µm). DSS treatment induced a pronounced thickening to 226.0 ± 19.9 µm, reflecting a 3.1‑fold increase, consistent with submucosal edema and inflammatory infiltration. Tofacitinib (131.2 ± 16.3 µm), sulfasalazine (155.2 ± 27.4 µm), and colon‑targeted tofacitinib beads (97.2 ± 16.7 µm) significantly reduced submucosal thickness by 42%, 31%, and 57%, respectively, compared with DSS (*P* < 0.05). The colon‑targeted formulation demonstrated the most pronounced effect, approaching control values. The placebo group (186.3 ± 26.0 µm) exhibited minimal improvement (18% reduction) and remained significantly thicker than DSS group (Fig. 6 (B)).

The thickness of the tunica muscularis in control rats was about 195.8 ± 42.3 µm, while tofacitinib alone produced comparable values (214.4 ± 41.8 µm). A marked increase in tunica muscularis thickness (571.8 ± 48.1 µm) was observed after induction of colitis by DSS, representing a 2.9‑fold (192%) increase relative to control. On the other hand, in the tofacitinib treated group, sulfasalazine and colon‑targeted tofacitinib beads groups, a significant (*P* < 0.05) decrease in the thickness was recorded compared with DSS treated group, with near-normal thickness observed in colon‑targeted tofacitinib beads group (209.2 ± 23.2 µm), corresponding to 63% reduction compared to DSS group. The placebo bead-treated group showed no significant decrease (512.3 ± 55.4 µm) in tunica muscularis thickness compared with the DSS group (Fig. 6 (C)).

### Significant reduction in elevated pro-inflammatory cytokines following treatment

**Fig. 7:**
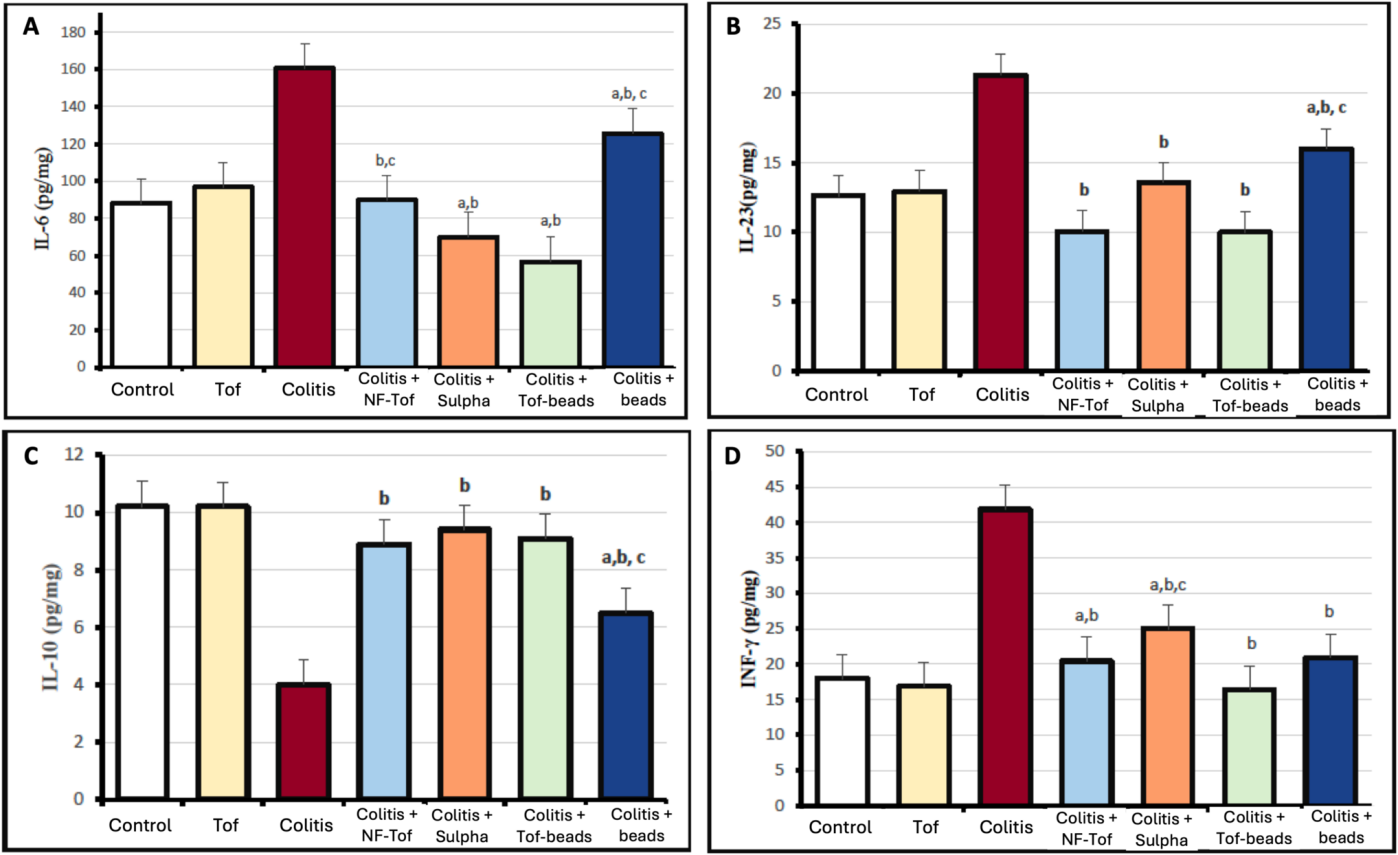
Effect of tofacitinib colon-targeted beads on the levels of inflammatory cytokines in in DSS-induced colitis (**A**) IL-6, (**B**) IL-23, (**C**) IL-10 and (**D**) INF-γ. Control group, Tof: Tofacitinib 20 mg/kg group; Colitis group: Colitis + NF-Tof, DSS 15% plus non-formulated tofacitinib 20 mg/kg group; Colitis + Sulpha, DSS 15% plus Sulphasalazine 500 mg/kg; Colitis + Tof-beads: DSS 15% plus tofacitinib colon-targeted beads 20 mg/kg group; and Colitis + beads: DSS 15% plus coated placebo beads group. Data are expressed as means ± SD. Statistical analysis was carried out using the Kruskal–Wallis test, followed by Dunn’s post‑hoc test. a: Statistically significant from control group values, (*P* < 0.05), b: Statistically significant from colitis group values, (*P* < 0.05), and c: Statistically significant from tofacitinib colon targeted beads group, (*P* < 0.05)

Treatment with tofacitinib (20 mg/kg) alone produced values comparable to those of the control group (96.84 ± 10.55 pg/mg vs. 88.14 ± 9.59 pg/mg). Administration of 15% DSS induced an approximately 1.8‑fold increase in colonic IL-6 compared with the control group (160.64 ± 19.84 pg/mg, *P* < 0.05). In contrast, sulfasalazine treatment reduced IL‑6 to 125.64 ± 17.64 pg/mg, and non‑formulated tofacitinib yielded 89.79 ± 12.37 pg/mg. Among DSS‑treated groups, the colon‑targeted tofacitinib beads achieved the greatest reduction, lowering IL‑6 level by approximately 65% relative to the DSS group (56.87 ± 17.10 pg/mg). The IL‑6 level in the placebo bead group (125.64 ± 17.64 pg/mg) remained largely unchanged compared with the DSS group (Fig. 7A).

A similar trend was observed for colonic IL-23 levels. Treatment with tofacitinib (20 mg/kg) alone produced values similar to those of the control group (12.95 ± 2.26 pg/mg vs. 12.63 ± 1.10 pg/mg). Administration of 15% DSS resulted in an approximately 1.7‑fold increase in colonic IL‑23 levels compared with the control group (21.33 ± 3.15 pg/mg, *P* < 0.05). In contrast, sulfasalazine treatment reduced IL‑23 to 13.58 ± 3.11 pg/mg, and non‑formulated tofacitinib lowered it further to 10.08 ± 1.00 pg/mg. Among the DSS‑treated groups, colon‑targeted tofacitinib beads produced the most pronounced reduction in IL‑23,, corresponding to an approximately 53% reduction relative to the DSS group (10.05 ± 4.50 pg/mg). This reduction was slightly greater but not statistically significant compared with the non‑formulated drug (10.05 ± 4.50 pg/mg vs. 10.08 ± 1.00 pg/mg). The IL‑23 level in the placebo bead group (16.00 ± 4.67 pg/mg) remained moderately elevated and was not significantly different from the DSS control (Fig. 7B).

Regarding colonic IL‑10 levels, treatment with tofacitinib (20 mg/kg) alone produced values comparable to the control (10.21 ± 1.90 pg/mg vs. 10.21 ± 1.00 pg/mg). Administration of 15% DSS resulted in an approximately 57% decrease in colonic IL‑10 compared with the control group (4.40 ± 0.59 pg/mg, *P* < 0.05). Among the DSS‑treated groups, sulfasalazine produced the highest numerical restoration of IL‑10 levels (9.49 ± 1.80 pg/mg), followed by the colon‑targeted tofacitinib beads (9.09 ± 1.60 pg/mg) and the non‑formulated drug (8.90 ± 1.50 pg/mg). Although all treatments significantly improved IL‑10 compared with the DSS group, no statistically significant differences were observed among treatment groups (*P* > 0.05). The placebo bead group showed only a modest improvement (6.20 ± 1.20 pg/mg) compared with the DSS group (Fig. 7C).

Analysis of colonic INF‑γ revealed that tofacitinib (20 mg/kg) alone produced values comparable to control (16.90 ± 0.90 pg/mg vs. 17.99 ± 2.90 pg/mg). Administration of 15% DSS caused a marked elevation in INF‑γ (41.86 ± 4.86 pg/mg), corresponding to an approximately 2.3‑fold increase relative to the control group (*P* < 0.05). Among DSS‑treated groups, all therapeutic interventions significantly reduced INF‑γ levels compared with the DSS group. Colon‑targeted tofacitinib beads produced the greatest suppression, lowering INF‑γ by approximately 61% relative to the DSS group (16.41 ± 1.90 pg/mg), and restoring values close to normal. Interestingly, INF‑γ levels were significantly lower in the colon‑targeted tofacitinib bead group than in the sulfasalazine‑treated group (*P* < 0.05). Moreover, the placebo bead group (20.90 ± 2.70 pg/mg) showed significantly lower INF‑γ levels than the DSS group (*P* < 0.05) (Fig. 7D).

### Enhanced colonic drug delivery with reduced systemic exposure with colon-targeted tofacitinib beads

**Fig. 8:**
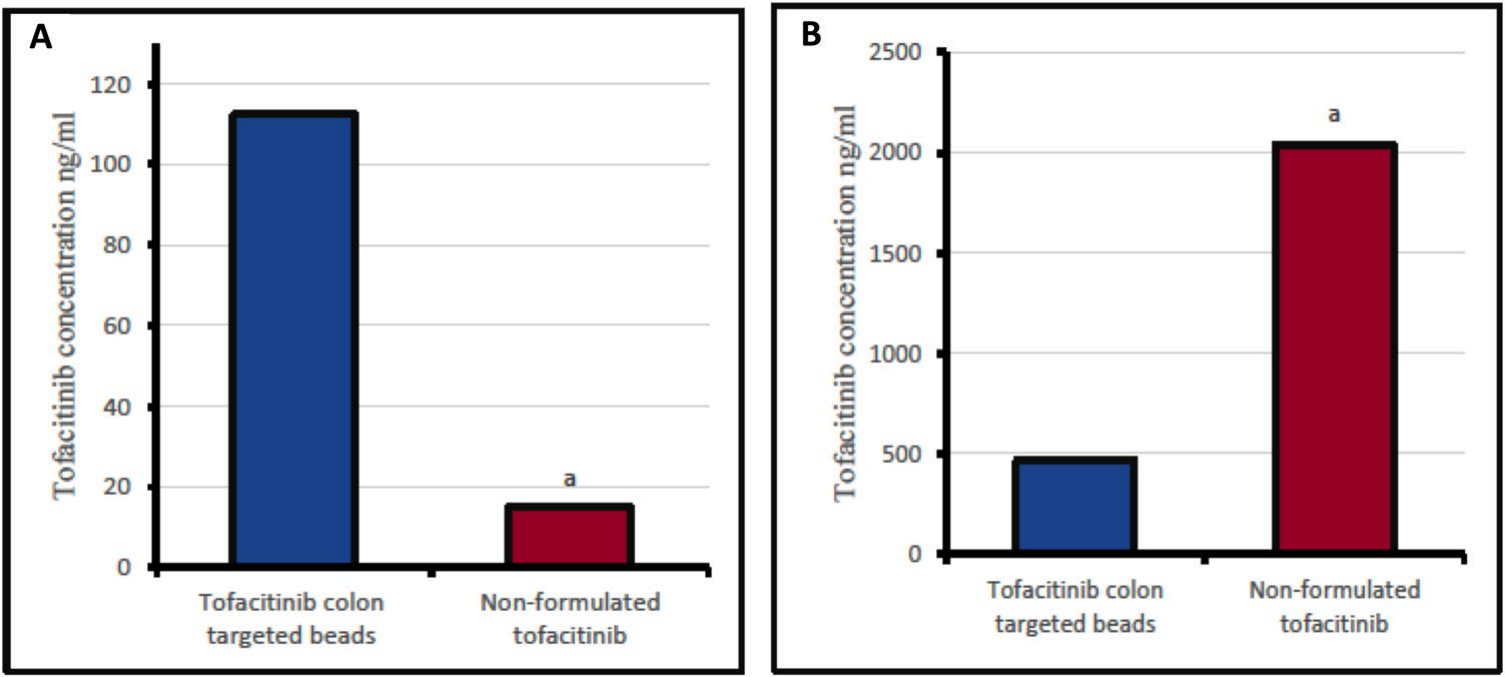
(**A**) Tofacitinib concentration in rat’s colon tissue. (**B**) Tofacitinib concentration in rat plasma. Data are expressed as median. Statistical analysis was carried out using a Mann–Whitney test. a: Significant change compared with corresponding tofacitinib colon targeted beads group values, (*P* < 0.05)

Colon‑targeted tofacitinib beads achieved markedly higher drug concentrations in colonic tissue (112.4 ng/mL) compared with non‑formulated tofacitinib group (15.0 ng/mL), representing an approximately 7.5‑fold increase (*P* < 0.05). Conversely, systemic exposure was significantly reduced, with plasma tofacitinib levels in the colon‑targeted bead group (467 ng/mL) being approximately 77% lower than those in the non‑formulated group (2041.7 ng/mL, *P* < 0.05). (Fig. 8A and B).

## Discussion

The study evaluates the therapeutic potential of colon‑targeted tofacitinib beads in DSS-induced colitis in rats, aiming to deliver tofacitinib specifically to the colon, thereby minimizing systemic absorption and reducing side effects. Accordingly, the principal contribution of this work lies in the comparative in vivo evaluation of colon‑targeted versus non‑formulated tofacitinib. The DSS-induced colitis model is a well-established and widely accepted approach that induces UC resembling the pathological characteristics of the human colon (Eichele and Kharbanda 2017). Although administration of 15% DSS by gavage is not a standard protocol, this approach was adopted following preliminary pilot experiments in which the conventional 5% DSS provided in drinking water (Cui et al. 2022) failed to induce reproducible clinical or histological signs of colitis. Because voluntary water intake varies substantially among animals and can result in inconsistent DSS exposure, a controlled gavage‑based method was implemented to ensure uniform dosing. Twice‑daily gastric administration of 15% DSS reliably induced mild‑to‑moderate colitis, characterized by colonic shortening, mucosal erythematous foci, mild weight loss, and stool discoloration, without evidence of severe systemic toxicity or dehydration. Validation of this modified model was performed by comparing the resulting pathological features with those described in established DSS colitis literature, confirming the presence of hallmark characteristics, including mucosal ulceration, crypt architectural distortion, goblet cell loss, and lamina propria leukocyte infiltration. These findings closely resemble classical DSS‑induced colitis patterns described by Chassaing (2014) and Eichele and Kharbanda (2017), indicating that this bolus‑administration approach produces physiologically relevant DSS colitis rather than nonspecific chemical injury.

The selected tofacitinib dose of 20 mg/kg twice daily (40 mg/kg/day) was based on previously published rodent studies evaluating JAK inhibition in experimental colitis. In particular, Beattie et al. (2017) demonstrated that oral tofacitinib at total daily doses of 30–45 mg/kg/day (10–15 mg/kg TID) produced significant reductions in disease activity index scores in the oxazolone colitis model. The total daily exposure used in the present study is therefore within the range previously shown to achieve effective colonic JAK inhibition in vivo. Although the disease model and dosing frequency differ, the pharmacodynamic requirements for JAK/STAT pathway suppression in rodent colitis models appear broadly comparable across studies. Importantly, the goal of the present work was not to evaluate systemic tofacitinib, but to compare it with a colon‑targeted formulation administered at the same nominal dose; using a sufficiently active systemic dose was therefore necessary to allow meaningful PK/PD comparison. The absence of treatment‑related systemic toxicity in the current study further indicates that this dose did not induce excessive systemic JAK inhibition in rats.

Beads were selected as drug delivery systems that release the drug in a specific body compartment due to their ability to provide controlled and targeted release. Currently, most available drug delivery systems use biodegradable, biocompatible, and natural biopolymers and are capable of rate-controlled drug release (Devi et al. 2010). Sodium alginate is widely used in various controlled release dosage forms due to its natural origin, biodegradable, mechanical strength, and hydrogel-forming properties. Alginates are naturally occurring polysaccharide polymers obtained from brown algae, consisting of two different monomeric units, β-D-mannuronic acid and α-L-guluronic acid (Andretto et al. 2023). In this study, tofacitinib beads were formulated using sodium alginate because of its ability to tailor the mechanical properties and degradability. Such beads are valued for their ease of fabrication and ability to assume various shapes (Tiwari et al. 2013). The beads were coated with Eudragit, a coating polymer that facilitates tofacitinib released at pH 7.1, thereby ensuring colon targeting. This release profile was confirmed in the *in vitro* release studies (Nikam et al. 2023). Although the encapsulation efficiency of approximately 48% is moderate for alginate‑based systems, the formulation demonstrated excellent stability over five months and, importantly, achieved superior *in vivo* colonic targeting with reduced systemic exposure, indicating that biological performance was not limited by encapsulation efficiency.

The *in vitro* release results showed that tofacitinib-coated beads released approximately 86% of tofacitinib within 6 h at pH 7.1, with only about 4% released during the first 2 h. The small amounts of tofacitinib released during this period may be related to drug loosely bound to the bead surface, which was released despite the Eudragit coating. The rapid release observed upon transition to pH 7.1 reflects dissolution of the Eudragit® S‑100 coating rather than uncontrolled burst release and is consistent with the intended colon‑triggered release mechanism. Similar results were reported by Manna et al. (2025), who developed a novel pH-sensitive Eudragit-coated bead containing a curcumin-mesalamine combination for colon-specific drug delivery. Within the first 2 h, 6% of curcumin and 8% of mesalamine were detected in a medium with pH of 1.2, while 85% of curcumin and 91.5% of mesalamine were released in pH 7.4 medium after 8 h (Mutalik et al. 2016).

DSS induced a significant shortening of colon length compared with the control group, consistent with earlier studies (Chassaing et al. 2014; Kwon et al. 2021). However, treatment with tofacitinib, sulphasalazine and colon targeted tofacitinib beads resulted in significant improvement in colon length. These findings underlined the anti-inflammatory effect of tofacitinib. DSS- induced shortening of the colon reflects the severity of inflammation, which was further confirmed by histopathological evaluation in the current study. DSS-induced crypt destruction, goblet cells loss, inflammatory cell infiltration, and mucosal ulceration. These findings documented the establishment of an acute colitis model in rats (Kwon et al. 2021). As expected, colon targeted tofacitinib beads markedly ameliorated inflammatory changes in the colon. The histopathological improvement correlates with colon length restoration. This finding is in agreement with previous findings (Andretto et al. 2023).

Chronic inflammation in UC can disrupt the intestinal epithelial barrier, leading to the mucosal ulceration and crypt distortion (Kaur and Goggolidou 2020; Jeruc 2022). Neutrophils invasion of crypt epithelium leads to cryptitis and crypt abscesses formation, disrupting normal crypt structure (AbdullGaffar et al. 2013). Repeated cycles of crypt destruction and aberrant regeneration result in architectural distortion, characterized by irregular, branched, dilated, and shortened crypts (Rubio et al. 2024).

A hallmark of UC pathology is the upregulation of pro-inflammatory cytokines, including IL-6, IL-23, and IFN-γ, which causes ongoing mucosal inflammation and delayed healing (Gyires et al. 2014). IL-6 and IL-23 support Th17 differentiation and activation, while IFN-γ promotes macrophage activation and amplifies inflammatory signaling (Neurath 2014). These cytokines perpetuate epithelial damage, crypt destruction dysregulation, which is the pathological basis for disease progression (Kobayashi et al. 2020).

As previously noted, tofacitinib is a JAK inhibitor. The JAK family consists of intracellular tyrosine kinases (TYK) JAK1, JAK2, JAK3, and TYK2. Cytokines essential for immune and stromal gut cell homeostasis, including IL-6, IL-10, IL-2, and IL-22, as well as those implicated in UC and Crohn’s disease pathologiy (IFN-γ, IL-12, IL-23, or IL-9), depend on JAK/STAT signaling, which is critical for restoring colonic homeostasis (Honap et al. 2024; Salas et al. 2020).

IL-6, a key pro-inflammatory cytokine, contributes significantly to the uncontrolled intestinal inflammation observed in UC. Elevated IL-6 in colitis group is explained by its role in increasing epithelial permeability, facilitating macrophage infiltration and exacerbates disease progression (Takač et al. 2014). Treatment with colon-targeted tofacitinib beads resulted in a significant reduction in IL-6 levels compared to both the colitis group and other treatment groups. This can be attributed to the drug’s ability to inhibit JAK1 phosphorylation, thereby suppressing IL-6 transcription. This finding aligns with the results of Yadav et al. (2022), who formulated tofacitinib-coated capsules in different doses, 1mg/kg and 10 mg/kg, significantly decreased colonic IL-6 concentrations compared with the untreated. IL-23 another key pro-inflammatory cytokine, was significantly reduced by colon-targeted tofacitinib beads. This effect may result from suppression of the IL-23/IL-17 axis, as previously shown in a study (Ghoreschi et al. 2011). Elevated IL‑23 correlates with disease severity in UC in human (Mohammadi et al. 2011).

In the present study, colon-targeted tofacitinib significantly increased IL-10 level in colonic tissue in the colitis model. IL-10 is a key anti-inflammatory cytokinin that suppresses cytokine production and antigen presentation. Specifically, IL-10 inhibits the synthesis of proinflammatory cytokines, including IL-1β, IL-6, as well as Th2 cell-derived cytokines such as IL-4 and IL-5 (Li and He 2004). A meta-analysis evaluating serum IL-10 in patients with UC reported elevated IL-10 level compared with healthy controls, with levels increasing in parallel with disease activity (Meng et al. 2019). Similarly, another study suggested that elevated IL-10 in UC may represent a compensatory mechanism aimed at counteracting excessive pro-inflammatory mediators responsible for inflammation and tissue injury (Leach et al. 1999). These findings are consistent with the present results and with previous experimental data from an oxazolone‑induced colitis model, in which IL‑10 levels were increased following tofacitinib treatment, accompanied by reductions in pro‑inflammatory cytokines including IL-5, IL-6, IL-13, and IL-17A (Gerlach et al. 2021).

IFN-γ is another key cytokine involved in intestinal inflammation and signals predominantly through activation of the JAK1/JAK2 pathway. The γ-chain cytokines, including IL-2, IL-4, IL-7, IL-9, IL-15, and IL-21, signal via JAK1/JAK3 and play important roles in adaptive immune regulation, including B-cell maturation and the differentiation of Th1, Th2, and Th17 cells (Hofmann et al. 2002). In our study, the level of INF-γ in colon tissue was reduced compared to the colitis group. This finding is consistent with the results of Gerlach et al. (2021), who reported that the levels of INF-γ and other pro-inflammatory cytokines, as well as the levels of IL-4, IFN-γ, and IL-17, were significantly inhibited in the tofacitinib-treated group compared to the untreated group.

The modest anti‑inflammatory effect observed in the placebo‑coated bead group may be attributed to the presence of sodium alginate, which possesses intrinsic protective and reparative properties in UC. Mirshafiey et al (2005) demonstrated that the sodium alginate significantly reduced IL-6 and tumor necrosis factor-ɑ (TNF-ɑ) levels in 4% acetic acid induced colitis rat’s model. Additionally, daily oral administration of sodium alginate (0.5% W/V solution) significantly reduced endoscopic colonic damage scores and improved histopathological outcomes, supporting its role in inhibiting mucosal injury.

In this study, tofacitinib concentrations were measured in both plasma and colon tissue. Animal treated with colon-targeted tofacitinib beads exhibited significantly lower plasma drug concentrations compared with those receiving commercial tofacitinib. Although full pharmacokinetic parameters (AUC, Cmax, Tmax) were not determined, the large and consistent differences observed at this time point were sufficient to demonstrate enhanced colonic targeting and markedly reduced systemic exposure. These findings are consistent with previous work in which ileocolonic‑targeted capsules formulated with Eudragit® S and resistant starch produced delayed peak plasma concentrations and significantly lower plasma Cmax values compared with tofacitinib solution or uncoated tablets, while achieving higher drug concentrations in colonic tissue (Yadav et al. 2022). In healthy male Lewis rats, these coated capsules produced delayed maximal plasma concentrations and significantly lower plasma Cmax values compared with tofacitinib solution and uncoated tablets. Coated capsules had significantly higher peak concentrations in the colon tissue than those exposed to untargeted formulations (solution and uncoated tablets), with a similar study (Yadav et al. 2022). Moreover, although systemic exposure was reduced in the colon‑targeted tofacitinib bead group, the present study did not evaluate hematological parameters, organ toxicity, or chronic safety; therefore, the safety profile of the formulation remains to be fully established.

The findings of this study have significant implications for the management of UC. A colon-targeted tofacitinib delivery system may represent a viable strategy to maximize local therapeutic efficacy while minimizing systemic adverse effects, including infections and thromboembolism, which currently limit clinical utility of systemic tofacitinib. This approach may be particularly beneficial for patients with extensive colonic involvement or those who are intolerant to systemic immunosuppression.

Furthermore, the success of this formulation underscores the broader applicability of pH-sensitive, polymer-coated drug delivery systems for localized treatment of inflammatory bowel disease. Future clinical trials are warranted to translate these preclinical findings into human application and to evaluate long-term outcomes, safety profiles, and patient-reported benefits. While further optimization of formulation parameters such as polymer concentration, crosslinking density, or coating thickness may enhance encapsulation efficiency, such optimization was beyond the scope of the present study, which focused on comparative in vivo performance.

## Conclusion

The present study demonstrates that colon-targeted delivery of tofacitinib using Eudragit S-100-coated sodium alginate beads provides significant therapeutic benefits in a DSS-induced colitis model. The formulation exhibited high stability, pH-dependent drug release, and superior colonic tissue targeting. Compared to conventional oral administration, the colon-targeted system significantly reduced disease severity, preserved colon architecture, modulated inflammatory cytokines profiles, and minimized systemic drug exposure. These findings support the potential of this delivery system as a safer and more effective alternative for tofacitinib administration in UC and warrant further investigation in pharmacokinetic and clinical studies.

## Data Availability

The authors confirm that the data supporting the findings of this study are available within the article.
